# Supplementary material for: PI3K and PINK1 Immunoexpression as Predictors of Survival in Patients Undergoing Resection of Brain Metastases from Lung Adenocarcinoma
Source: Int J Mol Sci. 2025 Mar 24;26(7):2945. doi: 10.3390/ijms26072945 (PMC11988690; doi:10.3390/ijms26072945)
Supplement: Supplementary file 1 [file ijms-26-02945-s001.zip › ijms-3527091-supplementary.pdf]

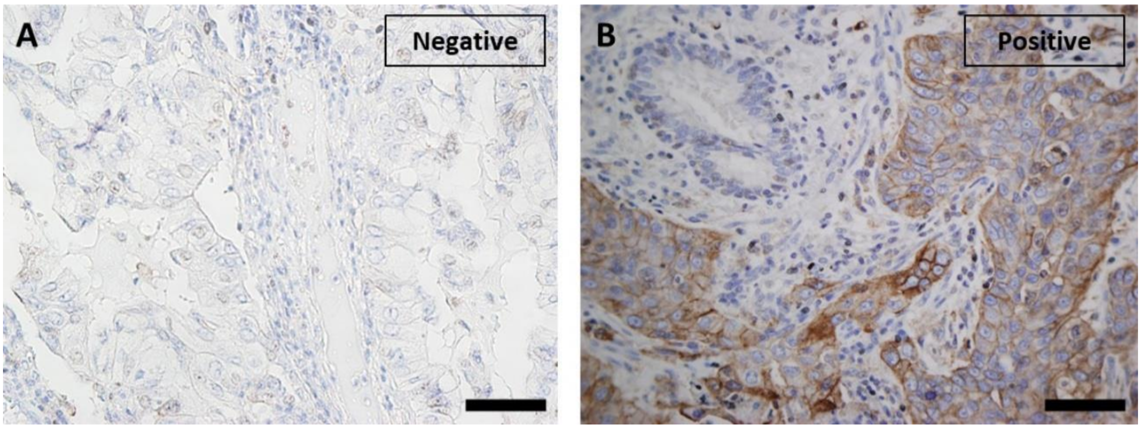

Supplemental Figure S1. PD-L1 Expression in Lung Adenocarcinomas. Representative images of PD-L1 staining are shown, illustrating negative and positive expression at 400× magnification. Scale bars in A and B = 40 μm.

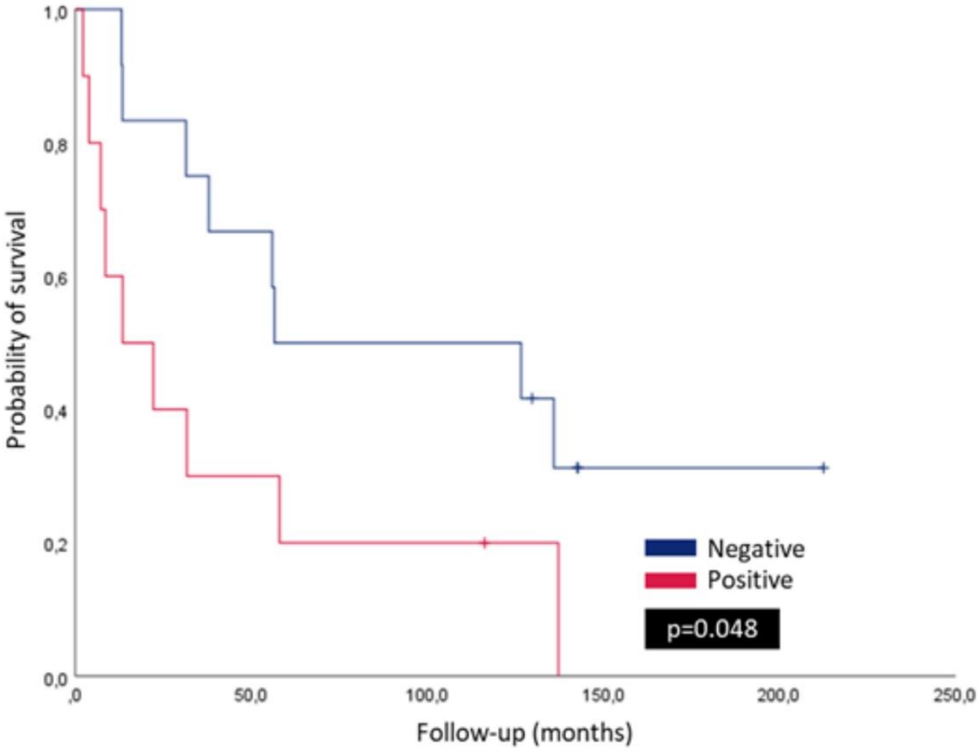

Supplemental Figure S2. Kaplan-Meier survival curves for PD-L1 expression. Patients with PD-L1-positive tumors had significantly reduced overall survival compared to PD-L1-negative patients (p = 0.048).
